# LIFELONG LEARNING: HOW HEALTH LITERACY SHIELDS OLDER ADULTS FROM THE MORTALITY RISKS OF LOWER EDUCATION

**DOI:** 10.1093/geroni/igae098.4231

**Published:** 2024-12-31

**Authors:** Hee Lee, Jieun Song, Jonghwa Do

**Affiliations:** The University of Alabama, Tuscaloosa, Alabama, United States; University of Wisconsin-Madison, Madison, Wisconsin, United States; University of Wisconsin-Madison, Madison, Wisconsin, United States

## Abstract

Research has demonstrated the significant impacts of education and health literacy (HL) on mortality risks. However, the combined effects of education and HL on mortality in older age, particularly using population samples, remain underexplored. This study investigates whether the association between education and mortality is moderated by varying levels of HL among older adults, using data from the Wisconsin Longitudinal Study (1957-2020). The analytic sample includes participants aged 60 and older at the 2010-2012 data collection (n=3,381, mean age=70.8). Mortality data were obtained from the National Death Index 2021 files, and HL was measured using the Newest Vital Sign (NVS) Health Literacy Score. Within this cohort, 531 respondents had limited HL, 893 had possibly limited HL, and 1,957 had adequate HL. Survival analysis using Cox Proportional Hazard models revealed that older adults with limited HL who completed only high school or some college education faced 22% and 49% higher mortality risks, respectively, compared to their peers with a college degree. Notably, high school graduates with adequate HL exhibited a 20% lower risk of mortality compared to those with a college degree but limited HL. These findings suggest that the adverse effects of lower education on mortality can be significantly mitigated by adequate health literacy, even in older age. This underscores the importance of interventions focused on enhancing health literacy, particularly among older adults with relatively lower educational attainment.

